# Crystal structure of (μ_2_-7-{[bis­(pyridin-2-ylmeth­yl)amino-1κ^3^*N*,*N*′,*N*′′]meth­yl}-5-chloro­quinolin-8-olato-2κ*N*;1:2κ^2^*O*)tri­chlorido-1κ*Cl*,2κ^2^*Cl*-dizinc(II)

**DOI:** 10.1107/S2056989024009782

**Published:** 2024-10-15

**Authors:** Koji Kubono, Kanata Tanaka, Keita Tani, Yukiyasu Kashiwagi

**Affiliations:** a Osaka Kyoiku University, 4-698-1 Asahigaoka Kashiwara Osaka 582-8582 Japan; b Osaka Research Institute of Industrial Science and Technology, 1-6-50 Morinomiya, Joto-ku, Osaka 536-8553, Japan; Tokyo University of Science, Japan

**Keywords:** crystal structure, dinuclear zinc(II) complex, 8-quinolinol, bis­(2-picol­yl)amine, C—H⋯Cl inter­actions

## Abstract

The title compound, [Zn_2_(C_22_H_18_ClN_4_O)Cl_3_], is a dinuclear zinc(II) complex with three chlorido ligands and one penta­dentate ligand containing quinolin-8-olato and bis­(pyridin-2-ylmeth­yl)amine groups. One of the two Zn^II^ atom adopts a tetra­hedral geometry and coordinates two chlorido ligands with chelate coord­ination of the N and O atoms of the quinolin-8-olato group in the ligand. The other Zn^II^ atom adopts a distorted trigonal–bipyramidal geometry, and coordinates one chlorido-O atom of the quinolin-8-olato group and three N atoms of the bis­(pyridin-2-ylmeth­yl)amine unit. In the crystal, two mol­ecules are associated through a pair of inter­molecular C—H⋯Cl hydrogen bonds, forming a dimer with an *R*_2_^2^(12) ring motif. Another inter­molecular C—H⋯Cl hydrogen bond forms a spiral *C*(8) chain running parallel to the [010] direction. The dimers are linked by these two inter­molecular C—H⋯Cl hydrogen bonds, generating a ribbon sheet structure in *ac* plane. Two other inter­molecular C—H⋯Cl hydrogen bonds form a *C*(7) chain along the *c*-axis direction and another *C*(7) chain generated by a *d*-glide plane. The mol­ecules are cross-linked through the four inter­molecular C—H⋯Cl hydrogen bonds to form a three-dimensional network.

## Abstract 2

The title compound is a dinuclear zinc(II) complex with three chlorido ligands and one penta­dentate ligand containing quinolin-8-olato and bis­(pyridin-2-ylmeth­yl)amine units. One Zn^II^ atom has a tetra­hedral coordination environment by two chlorido and chelate coordination of the N and O atoms of the quinolin-8-olato unit in the penta­dentate ligand, and the other has a distorted trigonal–bipyramidal coordination environment by one chlorido and four donating atoms except for the N atom of the quinolin-8-olato in the penta­dentate ligand. In the crystal, the mol­ecules are linked by four different kinds of inter­molecular C—H⋯Cl hydrogen bonds, forming a three-dimensional network structure.

## 1. Chemical context

Dinuclear metal complexes have received much attention due to their functional properties and many potential applications, such as active centre models of metalloproteins in bioinorganic chemistry (Wieghardt *et al.*, 1986 ▸), OLEDs (Pander *et al.*, 2023 ▸), chemosensors (Bazany-Rodríguez *et al.*, 2020 ▸), biosensors (Van der Heyden *et al.*, 2023 ▸), electrocatalysts (Raj *et al.*, 2023 ▸) and magnetic materials (Massoud *et al.*, 2015 ▸). With regard to the applications for chemosensors, fluore­scent anion probes based on metal complexes have been investigated, and dinuclear complex probes with high selectivity for target anions have been reported (Chen *et al.*, 2011 ▸; Mesquita *et al.*, 2016 ▸). We synthesized a penta­dentate ligand (HClqdpa) containing quinolin-8-ol (Hq) and bis­(pyridin-2-ylmeth­yl)amine [di-(2-picol­yl)amine, dpa] moieties, and its mononuclear Zn^II^ complex {7-{[bis­(pyridin-2-ylmeth­yl)amino-κ^3^*N*,*N′*,*N*′′]meth­yl}-5-chloro-quinolin-8-ol}di­bromido­zinc(II) [ZnBr^2^(HClqdpa)] to develop a fluorescent anion probe, and analysed their crystal structures (Kubono *et al.*, 2015 ▸, 2022 ▸). The Zn atom in this complex is five-coordinated by two bromido and three N atoms of the dpa group in the ligand. The Hq moiety in the ligand is not coordinated to the Zn atom. Therefore, a dinuclear complex, Zn:ligand = 2:1, can be formed by coordinating another zinc(II) ion to the Hq moiety in the mononuclear complex, since the O atom of quinolin-8-olato is able to bind with two metal ions through bridging coordination. Herein we report on the synthesis and crystal structure of the dizinc(II) title complex with HClqdpa and three chlorido atoms, Zn_2_Cl_3_(Clqdpa).
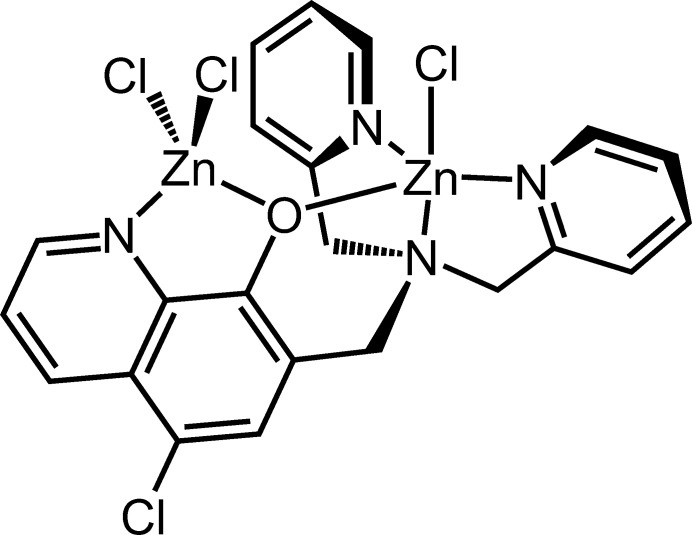


## 2. Structural commentary

**Figure 1:**
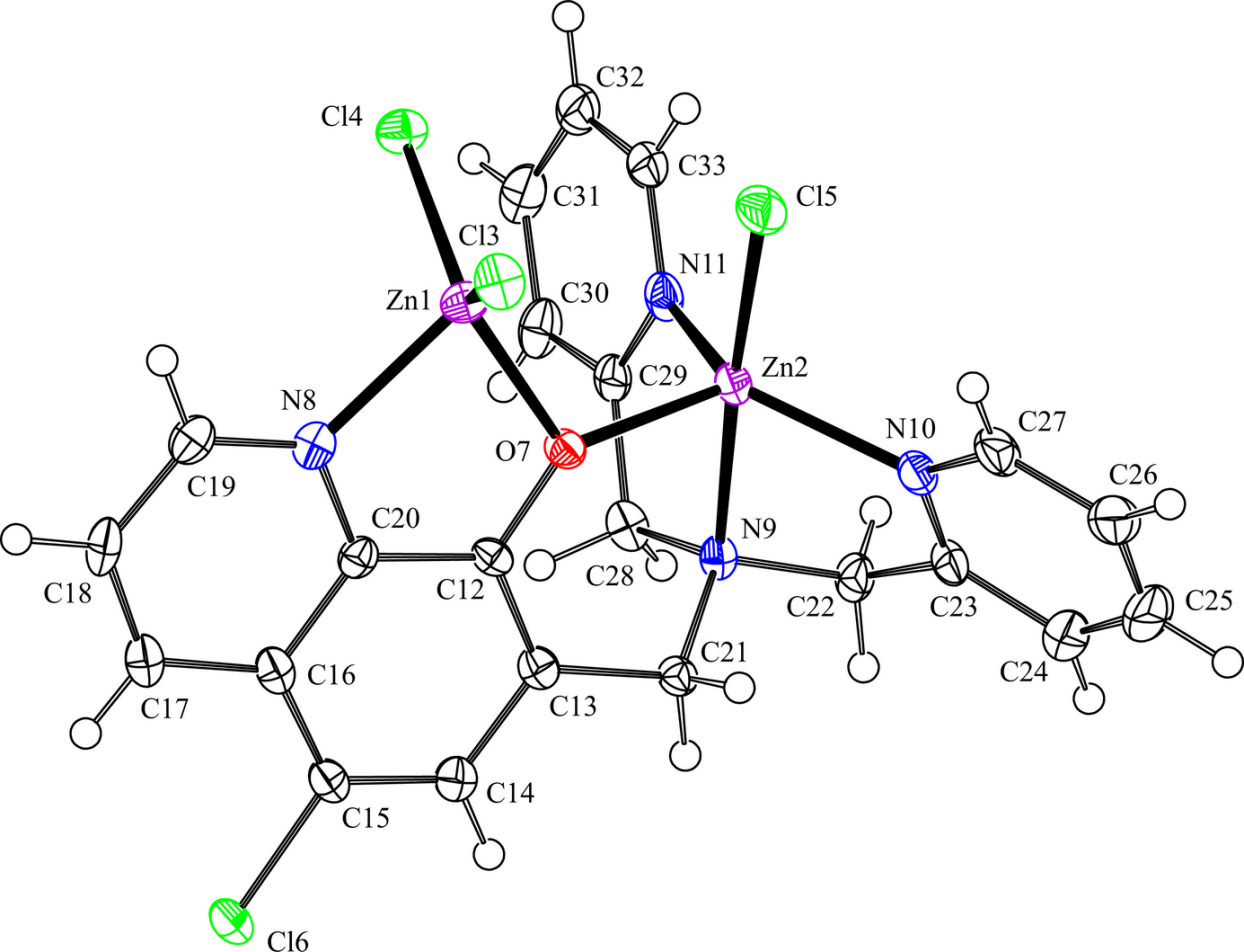
The mol­ecular structure of the title compound, with the atom labelling. Displacement ellipsoids are drawn at the 50% probability level. H atoms are represented by spheres of arbitrary radius.

The mol­ecular structure of the title compound is shown in Fig. 1 ▸. The mol­ecule is a dinuclear zinc(II) complex with three chlorido ligands and the penta­dentate ligand (Clqdpa) based on 5-chloro­quinolin-8-olato (Clq) and dpa groups. The Zn1 atom adopts distorted a tetra­hedral geometry and coordinates two chlorido ligands (Cl3 and Cl4) and the N8 atom and the O7 atom of the Clq unit in Clqdpa, forming a ZnCl_2_(Clq) unit. The Zn2 atom adopts a distorted trigonal–bipyramidal geometry and coordinates one chlorido ligand (Cl5), the O7 atom of the Clq unit and three N atoms (N9, N10 and N11) of the dpa group in the Clqdpa, forming a ZnCl(Clqdpa) unit, but the N8 atom in the Clq unit is not coordinated to the Zn2 atom. The four-coordinate geometry index, *τ*_4_ = [360°(*α* + *β*)]/141°, evaluated from the two largest angles (*α* < *β*), and has ideal values of 1 for a tetra­hedral and 0 for a square-planar geometry (Yang *et al.*, 2007 ▸), whereas the five-coordinate geometry index, *τ*_5_ = (*β* − *α*)/60, derived from the two largest angles (*α* < *β*) in a structure has ideal values of 1 for a trigonal–bipyramidal and of 0 for a square-pyramidal geometry (Addison *et al.*, 1984 ▸). In the title compound, *τ*_4_ for the Zn1 atom and *τ*_5_ for the Zn2 atom are equal to 0.861 and 0.832, respectively. In the tetra­hedral ZnCl_2_(Clq) unit, the Zn1—Cl3, Zn1—Cl4, Zn1—O7 and Zn1—N8 bond lengths are 2.2190 (10), 2.2241 (10), 2.019 (3) and 2.101 (3) Å, respectively (Table 1 ▸). In the trigonal–bipyramidal ZnCl(Clqdpa) unit, the O7 atom of the Clq unit and the N10 and N11 atoms of the pyridine rings in dpa group are equatorially bound to the Zn2 atom. The Zn2 atom is located 0.3188 (6) Å above the equatorial O7/N10/N11 plane. The axial positions are occupied by the Cl5 atom and the tertiary N9 atom of dpa group. The equatorial bond lengths Zn2—O7, Zn2—N10 and Zn2—N11 are 2.026 (3), 2.083 (3) and 2.052 (3) Å, respectively (Table 1 ▸), whereas the axial bonds Zn2—Cl5 and Zn2—N9 are 2.2897 (11) and 2.216 (3) Å, respectively, longer than those of equatorial bonds (Table 1 ▸). The axial angle N9—Zn2—Cl5 is 176.25 (8)°, and the equatorial angles range from 102.47 (11) to 126.31 (12)° (Table 1 ▸). The O atom in the Clq unit is bridged-coordinated with two Zn^II^ atoms. The Zn1—O7—Zn2 bond angle is 112.72 (12)° (Table 1 ▸). The mean planes of two pyridine rings in the dpa unit are not coplanar with the equatorial O7/N10/N11 plane of the trigonal bipyramid, but rather nearly perpendicular, the dihedral angles between the pyridine rings and the equatorial plane being 68.02 (19)° (for N10/C23–C27) and 83.38 (17)° (for N11/C29–C33). The dihedral angle between the two pyridine rings is 43.4 (2)°.

In contrast, the Zn atom in the related compound, ZnBr_2_(HClqdpa), adopts a distorted square-pyramidal geometry, and the dihedral angle between two pyridine rings is 15.84 (13)° (VAXNUH; Kubono *et al.*, 2022 ▸). In the other related compound, a dinuclear zinc(II) complex with the ligand having phenolato and two dpa units (RESSUH; Van der Heyden *et al.*, 2023 ▸), one Zn atom adopts a trigonal–bipyramidal geometry with one chlorido atom, and the other adopts a square-pyramidal geometry with an aqua O atom. Here the dihedral angles between two pyridine rings are 58.9 (3) and 9.6 (4)°, respectively, for the trigonal–bipyramidal and square pyramidal coordination geometries. The axial bond Zn—Cl length for the trigonal–bipyramidal Zn^II^ atom is 2.229 (2) Å, and the axial N—Zn—Cl bond angle is 177.4 (1)°, similar to those of the title compound.

The quinoline ring in the title compound is slightly bent with an r.m.s. deviation of 0.017 (4) Å. In the quinoline ring, the largest deviation from the mean plane is 0.022 (4) Å for carbon atom C14. The quinoline plane subtends dihedral angles of 76.81 (15) and 56.29 (17)° with the two pyridine rings.

## 3. Supra­molecular features

**Figure 2:**
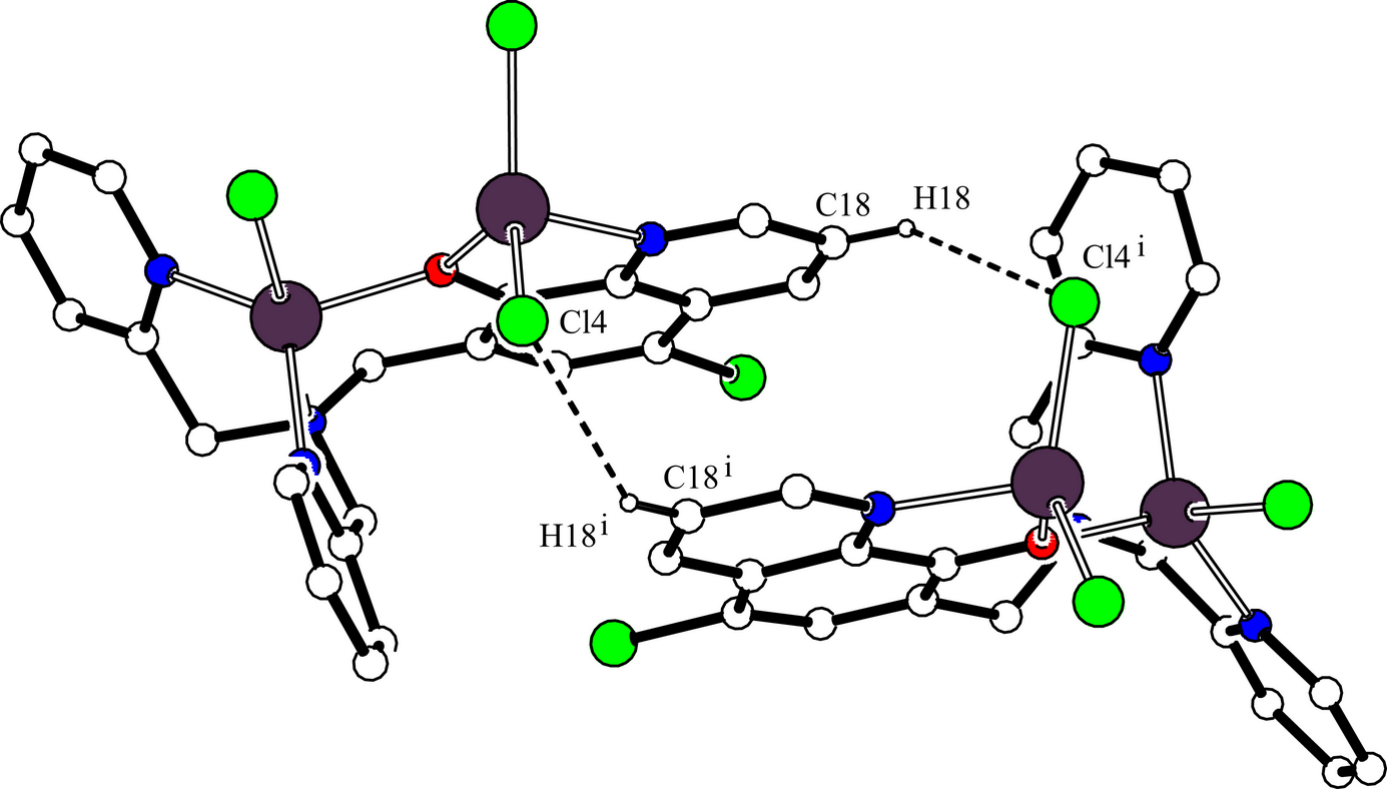
The twofold symmetric dimeric structure of the title compound. The inter­molecular C18—H18⋯Cl4^i^ hydrogen bonds are shown as dashed lines. H atoms not involved in these inter­actions have been omitted for clarity. [Symmetry code: (i) −*x* + 1, −*y* + 1, *z*.]

**Figure 3:**
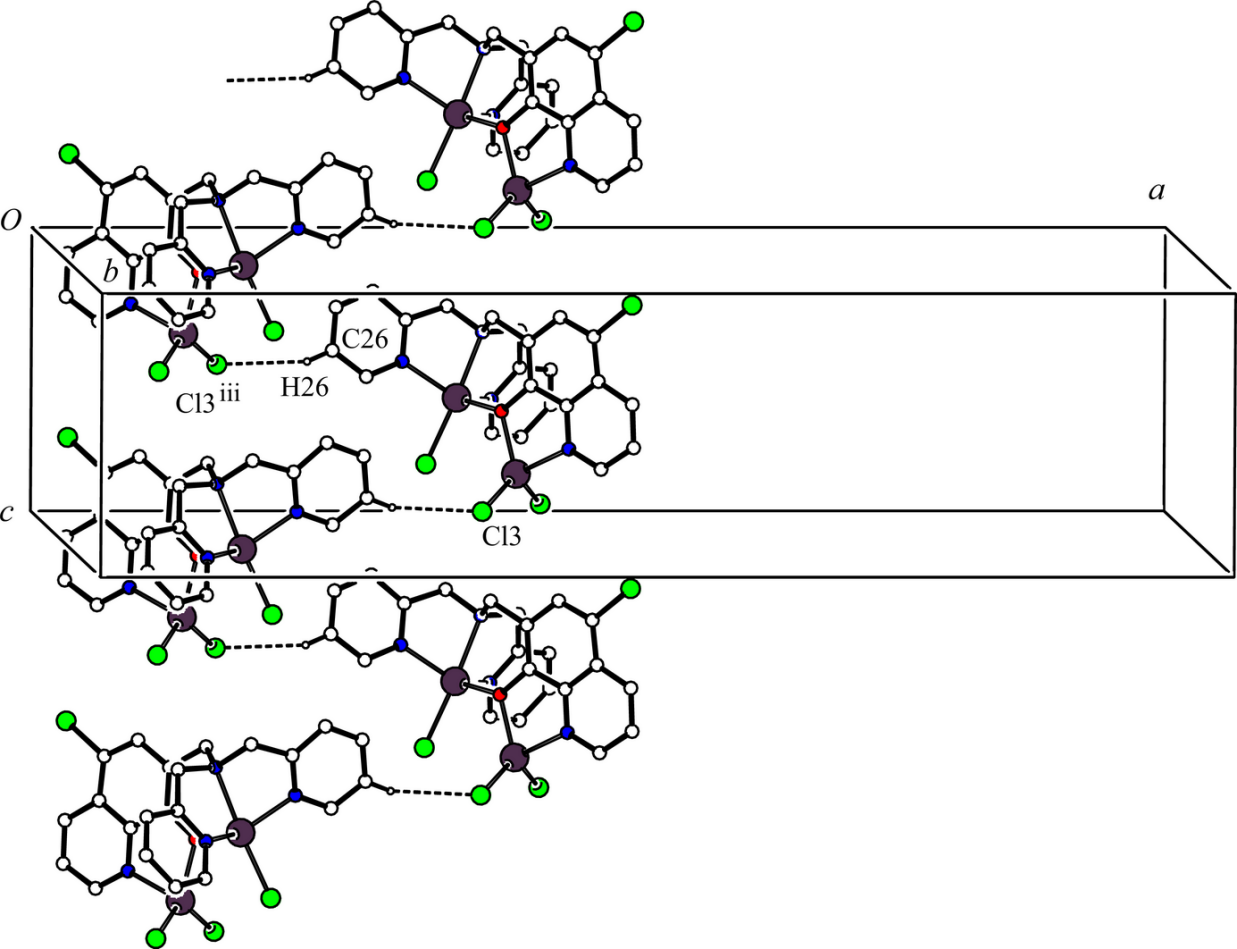
A portion of the crystal packing of the title compound showing the spiral *C*(8) chain formed *via* a 2_1_ screw axis. The inter­molecular C26—H26⋯Cl3^iii^ hydrogen bonds are shown as dashed lines. H atoms not involved in the inter­actions were omitted for clarity. [Symmetry code: (iii) −*x* + 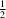, −*y* + 1, *z* − 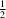.]

**Figure 4:**
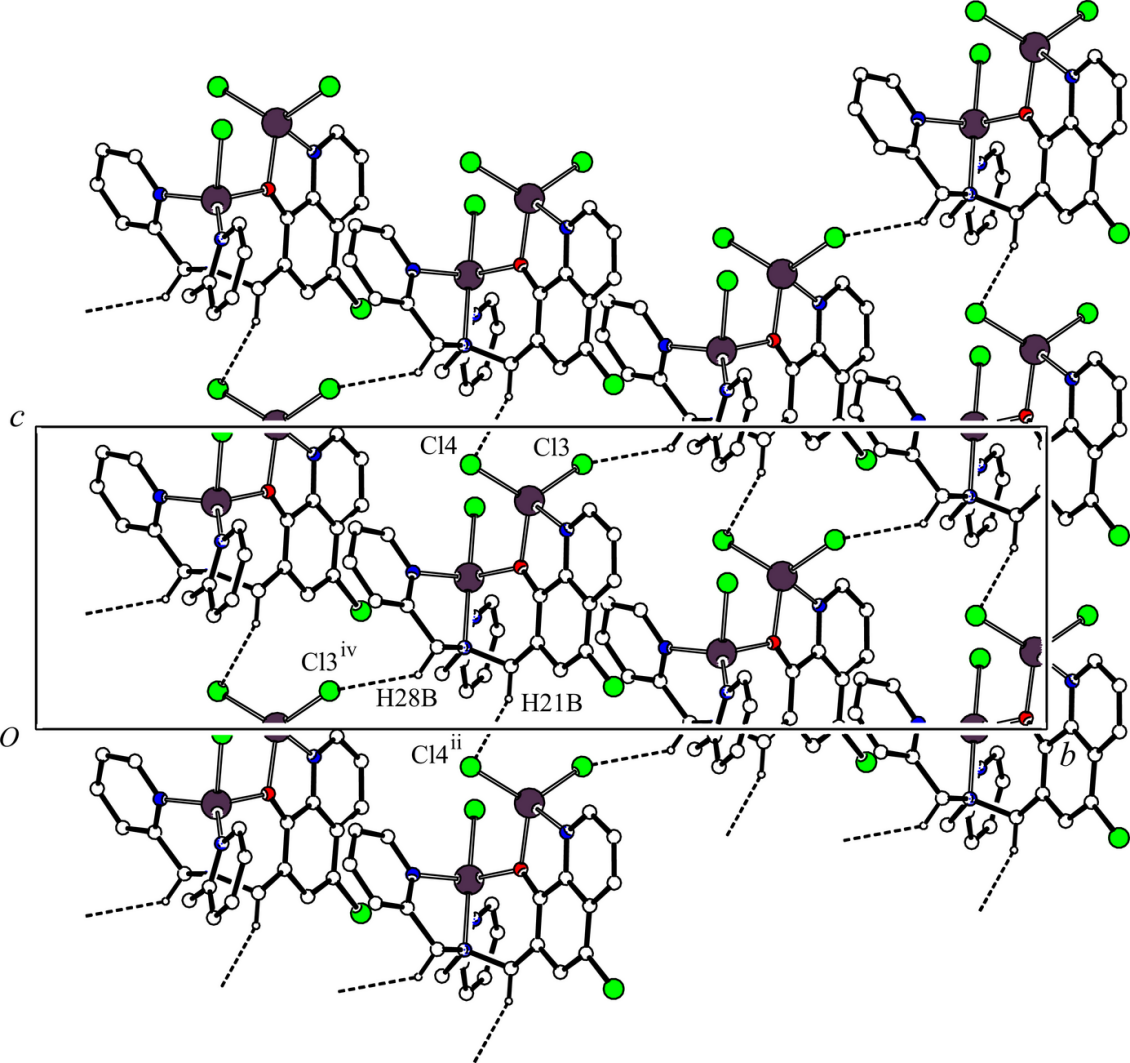
A packing diagram of the title compound viewed along the *a* axis, showing the two-dimensional network structure. The inter­molecular C21—H21*B*⋯Cl4^ii^ and C28—H28*B*⋯Cl3^iv^ hydrogen bonds are shown as dashed lines. H atoms not involved in the inter­actions have been omitted for clarity. [symmetry codes: (ii) *x*, *y*, *z* − 1; (iv) −*x* + 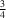, *y* − 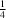, *z* − 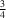.]

In the crystal, two mol­ecules are associated through a pair of inter­molecular C—H⋯Cl hydrogen bonds [C18—H18⋯Cl4^i^; symmetry code: (i) −*x* + 1, −*y* + 1, *z*; Table 2 ▸], forming a dimer with an 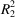
(12) ring motif by a two-fold axis (Fig. 2 ▸). Another inter­molecular C—H⋯Cl hydrogen bond is observed [C26—H26⋯Cl3^iii^; symmetry code: (iii) −*x* + 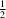, −*y* + 1, *z* − 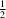; Table 2 ▸], which forms a spiral *C*(8) chain running parallel to the [010] direction by a 2_1_ screw axis (Fig. 3 ▸). The dimers with twofold symmetry are linked to each other by the inter­molecular C26—H26⋯Cl3^iii^ hydrogen bonds generating a ribbon sheet structure in the *ac* plane. The inter­molecular C21—H21*B*⋯Cl3^ii^ and C28—H28*B*⋯Cl4^iv^ hydrogen bond [symmetry code: (ii) *x*, *y*, *z* − 1; (iv) −*x* + 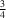, *y* − 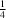, *z* − 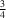; Table 2 ▸] form a *C*(7) chain along the *c*-axis direction and another *C*(7) chain generated by a *d*-glide plane, respectively. The mol­ecules are linked by these two inter­molecular C—H⋯Cl hydrogen bonds, generating a sheet structure in the *bc* plane (Fig. 4 ▸). Therefore, the mol­ecules are cross-linked through the four inter­molecular C—H⋯Cl hydrogen bonds to form a three-dimensional network.

## 4. Database survey

A search of the Cambridge Structural Database (CSD, Version 2024.1.0, update of March 2024; Groom *et al.*, 2016 ▸) using *ConQuest* (Bruno *et al.*, 2002 ▸) for Zn^II^ complexes with the [bis­(pyridin-2-ylmeth­yl)amino]­methyl fragment as ligand gave 641 hits, and among those 46 hits with one chlorido ligand. Zn^II^ complexes with the 2-[{bis­(pyridin-2-ylmeth­yl)amino}­meth­yl]phenolato fragment gave 133 hits and among those 15 hits for five-coordinated structures with one chlorido ligand. Of these 15 analogues, 10 structures have a trigonal–bipyramidal geometry whose apical positions are occupied by the Cl atom and the tertiary N atom, and five structures have a square-pyramidal geometry. The dihedral angles between the two pyridine rings range from 42.1 (8) to 77.79 (16)° in the ten trigonal–bipyramidal structures, while those of the five square-pyramidal structures range from 8.1 (5) to 36.29 (9)°.

A search for Zn^II^ complexes with the quinolin-8-olato fragment as ligand gave 244 hits and among these, dinuclear Zn^II^ complexes gave 71 hits. All the 71 structures contain multiple quinolin-8-olato moieties. Two structures among these 71 analogues are polymorphs of the Zn^II^ complex with the ligand in which the Cl atom of HClqdpa is replaced with an H atom, bis­(μ-7-({bis­[(pyridin-2-yl)meth­yl]amino}­meth­yl)quinolin-8-olato)dizinc(II) bis­(tetra­phenyl­borate). The rel­ated complex is a Zn:ligand = 2:2 dimeric dinuclear structure with the quinolin-8-olato O atom bridging two Zn^II^ ions (FEDTUH and FEDTOB; Kong *et al.*, 2022 ▸). In addition, a search for di­chlorido Zn^II^ complexes with the quinolin-8-olato fragment gave nine hits. Of these nine analogues, seven structures are Zn:ligand = 1:1 mononuclear complexes, one structure is a co-crystal with a 1:1 mononuclear complex and 1:2 dinuclear complex, and the last structure is a 1:1 *catena* complex. Therefore, the crystal structure of a 2:1 dinuclear Zn^II^ complex with a singular quinolin-8-olato has not been reported.

## 5. Synthesis and crystallization

The HClqdpa ligand was prepared by a reported method (Kubono *et al.*, 2015 ▸). HClqdpa (39.1 mg, 0.100 mmol) was dissolved in 15 mL of hot aceto­nitrile. Then a solution of zinc(II) chloride (34.1 mg, 0.250 mmol) in 15 mL of hot aceto­nitrile was added to the ligand solution. The mixture was stirred for 20 min at 333 K. After removal of the solvent at room temperature in the air for one week, yellow crystals of the title compound were obtained (yield 68.7%). ^1^H NMR (CD_3_SOCD_3_, 400 MHz): *δ* = 4.14 (*s*, 2H), 4.54, 4.79 (*ABq*, *J* = 16.8 Hz, 4H), 6.98–7.01 (*dd*, *J* = 8.0 Hz, *J* = 4.8 Hz, 2H), 7.32–7.33 (*d*, *J* = 4.8 Hz, 2H), 7.44–7.47 (*dd*, *J* = 8.8 Hz, *J* = 4.8 Hz, 1H), 7.68–7.70 (*dd*, *J* = 8.8 Hz, *J* = 4.4 Hz, 2H), 7.81 (*s*, 1H), 7.93-7.95 (*t*, *J* = 8.0 Hz, 2H), 8.23-8.24 (*d*, *J* = 4.4 Hz, 1H), 8,42–8.45 (*d*, *J* = 8.8 Hz, 1H).

## 6. Refinement

Crystal data, data collection and structure refinement details are summarized in Table 3 ▸. All H atoms bound to carbon were positioned geometrically and refined using a riding model, with C—H = 0.95–0.99 Å and *U*_iso_(H) = 1.2*U*_eq_(C).

## Supplementary Material

Crystal structure: contains datablock(s) I. DOI: 10.1107/S2056989024009782/jp2012sup1.cif

Structure factors: contains datablock(s) I. DOI: 10.1107/S2056989024009782/jp2012Isup2.hkl

CCDC reference: 2389039

Additional supporting information:  crystallographic information; 3D view; checkCIF report

## Figures and Tables

**Table 1:** Selected geometric parameters (Å, °)

Zn1—Cl3	2.2190 (10)	Zn2—O7	2.026 (3)
Zn1—Cl4	2.2241 (10)	Zn2—N9	2.216 (3)
Zn1—O7	2.019 (3)	Zn2—N10	2.083 (3)
Zn1—N8	2.101 (3)	Zn2—N11	2.052 (3)
Zn2—Cl5	2.2897 (11)		
			
Cl3—Zn1—Cl4	121.76 (4)	O7—Zn2—N11	102.47 (11)
O7—Zn1—Cl3	116.11 (8)	N9—Zn2—Cl5	176.25 (8)
O7—Zn1—Cl4	116.86 (8)	N10—Zn2—Cl5	98.31 (10)
O7—Zn1—N8	80.68 (11)	N10—Zn2—N9	77.99 (12)
N8—Zn1—Cl3	103.09 (9)	N11—Zn2—Cl5	103.95 (10)
N8—Zn1—Cl4	107.56 (9)	N11—Zn2—N9	78.95 (12)
O7—Zn2—Cl5	94.38 (8)	N11—Zn2—N10	126.31 (12)
O7—Zn2—N9	87.24 (10)	Zn1—O7—Zn2	112.72 (12)
O7—Zn2—N10	123.92 (11)		

**Table 2:** Hydrogen-bond geometry (Å, °)

*D*—H⋯*A*	*D*—H	H⋯*A*	*D*⋯*A*	*D*—H⋯*A*
C18—H18⋯Cl4^i^	0.95	2.77	3.537 (4)	139
C21—H21*B*⋯Cl4^ii^	0.99	2.69	3.584 (4)	150
C26—H26⋯Cl3^iii^	0.95	2.83	3.657 (4)	146
C28—H28*B*⋯Cl3^iv^	0.99	2.77	3.507 (4)	132

Symmetry codes: (i) 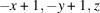; (ii) 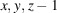; (iii) 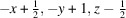; (iv) 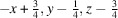.

**Table 3:** Experimental details

Crystal data	
Chemical formula	[Zn_2_(C_22_H_18_ClN_4_O)Cl_3_]
*M*_r_	626.98
Crystal system, space group	Orthorhombic, *Fdd*2
Temperature (K)	100
*a*, *b*, *c* (Å)	35.5812 (4), 29.7570 (3), 8.8942 (1)
*V* (Å^3^)	9417.09 (18)
*Z*	16
Radiation type	Cu *K*α
μ (mm^−1^)	6.89
Crystal size (mm)	0.37 × 0.15 × 0.06

Data collection	
Diffractometer	XtaLAB Synergy, Dualflex, HyPix
Absorption correction	Multi-scan (*CrysAlis PRO*; Rigaku OD, 2023 ▸)
*T*_min_, *T*_max_	0.318, 1.000
No. of measured, independent and observed [*I* > 2σ(*I*)] reflections	12774, 3507, 3445
*R*_int_	0.037
(sin θ/λ)_max_ (Å^−1^)	0.633

Refinement	
*R*[*F*^2^ > 2σ(*F*^2^)], *wR*(*F*^2^), *S*	0.028, 0.073, 1.04
No. of reflections	3507
No. of parameters	298
No. of restraints	1
H-atom treatment	H-atom parameters constrained
Δρ_max_, Δρ_min_ (e Å^−3^)	0.63, −0.36
Absolute structure	Flack *x* determined using 942 quotients [(*I*^+^)−(*I*^−^)]/[(*I*^+^)+(*I*^−^)] (Parsons *et al.*, 2013 ▸)
Absolute structure parameter	0.004 (17)

Computer programs: *CrysAlis PRO* (Rigaku OD, 2023 ▸), *SHELXT2018/2* (Sheldrick, 2015*a* ▸), *SHELXL2018/3* (Sheldrick, 2015*b* ▸), *PLATON* (Spek, 2020 ▸) and *OLEX2* (Dolomanov *et al.*, 2009 ▸).

## supplementary crystallographic information

### (µ2-7-{[Bis(pyridin-2-ylmethyl)amino-1κ3N,N',N'']methyl}-5-chloroquinolin-8-olato-2κN;1:2κ2O)trichlorido-1κCl,2κ2Cl-dizinc(II) Crystal data

**Table:**

[Zn_2_(C_22_H_18_ClN_4_O)Cl_3_]	*D*_x_ = 1.769 Mg m^−^^3^
*M**_r_* = 626.98	Cu *K*α radiation, λ = 1.54184 Å
Orthorhombic, *F**d**d*2	Cell parameters from 9909 reflections
*a* = 35.5812 (4) Å	θ = 3.9–76.7°
*b* = 29.7570 (3) Å	µ = 6.89 mm^−^^1^
*c* = 8.8942 (1) Å	*T* = 100 K
*V* = 9417.09 (18) Å^3^	Block, yellow
*Z* = 16	0.37 × 0.15 × 0.06 mm
*F*(000) = 5024	

### (µ2-7-{[Bis(pyridin-2-ylmethyl)amino-1κ3N,N',N'']methyl}-5-chloroquinolin-8-olato-2κN;1:2κ2O)trichlorido-1κCl,2κ2Cl-dizinc(II) Data collection

**Table:**

XtaLAB Synergy, Dualflex, HyPix diffractometer	3507 independent reflections
Radiation source: micro-focus sealed X-ray tube, PhotonJet (Cu) X-ray Source	3445 reflections with *I* > 2σ(*I*)
Mirror monochromator	*R*_int_ = 0.037
Detector resolution: 10.0000 pixels mm^-1^	θ_max_ = 77.3°, θ_min_ = 3.9°
ω scans	*h* = −44→39
Absorption correction: multi-scan (CrysAlisPro; Rigaku OD, 2023)	*k* = −36→36
*T*_min_ = 0.318, *T*_max_ = 1.000	*l* = −10→7
12774 measured reflections	

### (µ2-7-{[Bis(pyridin-2-ylmethyl)amino-1κ3N,N',N'']methyl}-5-chloroquinolin-8-olato-2κN;1:2κ2O)trichlorido-1κCl,2κ2Cl-dizinc(II) Refinement

**Table:**

Refinement on *F*^2^	Hydrogen site location: inferred from neighbouring sites
Least-squares matrix: full	H-atom parameters constrained
*R*[*F*^2^ > 2σ(*F*^2^)] = 0.028	*w* = 1/[σ^2^(*F*_o_^2^) + (0.053*P*)^2^] where *P* = (*F*_o_^2^ + 2*F*_c_^2^)/3
*wR*(*F*^2^) = 0.073	(Δ/σ)_max_ = 0.001
*S* = 1.04	Δρ_max_ = 0.63 e Å^−^^3^
3507 reflections	Δρ_min_ = −0.35 e Å^−^^3^
298 parameters	Absolute structure: Flack *x* determined using 942 quotients [(*I*^+^)-(*I*^-^)]/[(*I*^+^)+(*I*^-^)] (Parsons *et al.*, 2013)
1 restraint	Absolute structure parameter: 0.004 (17)
Primary atom site location: dual	

### (µ2-7-{[Bis(pyridin-2-ylmethyl)amino-1κ3N,N',N'']methyl}-5-chloroquinolin-8-olato-2κN;1:2κ2O)trichlorido-1κCl,2κ2Cl-dizinc(II) Special details

**Table:**

Geometry. All esds (except the esd in the dihedral angle between two l.s. planes) are estimated using the full covariance matrix. The cell esds are taken into account individually in the estimation of esds in distances, angles and torsion angles; correlations between esds in cell parameters are only used when they are defined by crystal symmetry. An approximate (isotropic) treatment of cell esds is used for estimating esds involving l.s. planes.
Refinement. 1. Fixed Uiso At 1.2 times of: All C(H) groups, All C(H,H) groups 2.a Secondary CH2 refined with riding coordinates: C21(H21A,H21B), C22(H22A,H22B), C28(H28A,H28B) 2.b Aromatic/amide H refined with riding coordinates: C14(H14), C17(H17), C18(H18), C19(H19), C24(H24), C25(H25), C26(H26), C27(H27), C30(H30), C31(H31), C32(H32), C33(H33)

### (µ2-7-{[Bis(pyridin-2-ylmethyl)amino-1κ3N,N',N'']methyl}-5-chloroquinolin-8-olato-2κN;1:2κ2O)trichlorido-1κCl,2κ2Cl-dizinc(II) Fractional atomic coordinates and isotropic or equivalent isotropic displacement parameters (Å^2^)

**Table:**

	*x*	*y*	*z*	*U*_iso_*/*U*_eq_	
Zn1	0.39776 (2)	0.48827 (2)	0.75531 (6)	0.01944 (13)	
Zn2	0.34873 (2)	0.42831 (2)	0.50092 (6)	0.01706 (12)	
Cl3	0.36491 (2)	0.54041 (3)	0.87591 (12)	0.0251 (2)	
Cl4	0.42290 (2)	0.42993 (3)	0.87494 (11)	0.02305 (19)	
Cl5	0.32149 (3)	0.43405 (3)	0.73337 (11)	0.0276 (2)	
Cl6	0.49423 (3)	0.57151 (3)	0.14036 (12)	0.0263 (2)	
O7	0.38518 (7)	0.47959 (8)	0.5358 (3)	0.0176 (5)	
N8	0.44197 (8)	0.52514 (10)	0.6589 (4)	0.0197 (6)	
N9	0.37150 (8)	0.42401 (9)	0.2695 (4)	0.0166 (6)	
N10	0.30023 (8)	0.43364 (10)	0.3710 (4)	0.0204 (6)	
N11	0.38276 (8)	0.37289 (10)	0.5185 (4)	0.0209 (6)	
C12	0.40978 (10)	0.49869 (11)	0.4411 (4)	0.0155 (7)	
C13	0.40764 (10)	0.49602 (12)	0.2861 (4)	0.0178 (7)	
C14	0.43408 (10)	0.51881 (12)	0.1959 (4)	0.0193 (7)	
H14	0.432327	0.516595	0.089588	0.023*	
C15	0.46223 (9)	0.54408 (12)	0.2582 (5)	0.0194 (7)	
C16	0.46627 (10)	0.54784 (12)	0.4154 (5)	0.0187 (7)	
C17	0.49488 (10)	0.57201 (12)	0.4911 (5)	0.0223 (8)	
H17	0.513079	0.588346	0.435234	0.027*	
C18	0.49622 (11)	0.57178 (14)	0.6438 (5)	0.0257 (8)	
H18	0.515411	0.587826	0.695187	0.031*	
C19	0.46909 (11)	0.54770 (13)	0.7252 (4)	0.0233 (8)	
H19	0.470359	0.547689	0.831868	0.028*	
C20	0.43993 (9)	0.52418 (11)	0.5065 (4)	0.0171 (7)	
C21	0.37708 (9)	0.46999 (12)	0.2078 (4)	0.0167 (7)	
H21A	0.353198	0.486860	0.216492	0.020*	
H21B	0.383334	0.467646	0.099665	0.020*	
C22	0.34150 (10)	0.40164 (13)	0.1832 (4)	0.0213 (8)	
H22A	0.341707	0.368970	0.204282	0.026*	
H22B	0.345763	0.406013	0.074185	0.026*	
C23	0.30396 (11)	0.42140 (12)	0.2274 (5)	0.0224 (8)	
C24	0.27451 (12)	0.42577 (17)	0.1257 (6)	0.0345 (10)	
H24	0.277287	0.416743	0.023812	0.041*	
C25	0.24095 (13)	0.4437 (2)	0.1776 (6)	0.0465 (14)	
H25	0.220416	0.447312	0.110362	0.056*	
C26	0.23711 (12)	0.45650 (19)	0.3269 (6)	0.0388 (12)	
H26	0.214191	0.468718	0.363367	0.047*	
C27	0.26757 (10)	0.45089 (14)	0.4205 (5)	0.0279 (9)	
H27	0.265455	0.459461	0.523040	0.034*	
C28	0.40620 (10)	0.39697 (12)	0.2715 (5)	0.0200 (7)	
H28A	0.428240	0.417275	0.270432	0.024*	
H28B	0.407240	0.378119	0.179901	0.024*	
C29	0.40814 (10)	0.36714 (12)	0.4087 (4)	0.0188 (7)	
C30	0.43723 (11)	0.33591 (13)	0.4239 (5)	0.0260 (9)	
H30	0.455288	0.332306	0.346154	0.031*	
C31	0.43942 (13)	0.31046 (14)	0.5526 (5)	0.0302 (9)	
H31	0.459074	0.289150	0.564504	0.036*	
C32	0.41274 (12)	0.31607 (13)	0.6651 (5)	0.0290 (9)	
H32	0.413594	0.298538	0.754302	0.035*	
C33	0.38493 (11)	0.34785 (13)	0.6438 (5)	0.0251 (8)	
H33	0.366696	0.352139	0.720489	0.030*	

### (µ2-7-{[Bis(pyridin-2-ylmethyl)amino-1κ3N,N',N'']methyl}-5-chloroquinolin-8-olato-2κN;1:2κ2O)trichlorido-1κCl,2κ2Cl-dizinc(II) Atomic displacement parameters (Å^2^)

**Table:**

	*U*^11^	*U*^22^	*U*^33^	*U*^12^	*U*^13^	*U*^23^
Zn1	0.0234 (2)	0.0193 (2)	0.0156 (2)	0.00292 (19)	0.0010 (2)	−0.0012 (2)
Zn2	0.0142 (2)	0.0171 (2)	0.0199 (2)	−0.00157 (16)	0.00064 (19)	0.0001 (2)
Cl3	0.0245 (4)	0.0201 (4)	0.0305 (5)	0.0033 (3)	0.0029 (4)	−0.0064 (4)
Cl4	0.0237 (4)	0.0232 (4)	0.0222 (4)	0.0060 (3)	0.0023 (4)	0.0021 (3)
Cl5	0.0248 (4)	0.0353 (5)	0.0227 (5)	−0.0029 (4)	0.0062 (4)	−0.0006 (4)
Cl6	0.0228 (4)	0.0313 (5)	0.0249 (5)	−0.0102 (3)	0.0021 (4)	0.0041 (4)
O7	0.0194 (12)	0.0174 (12)	0.0159 (13)	−0.0031 (9)	0.0015 (9)	−0.0010 (10)
N8	0.0196 (14)	0.0195 (15)	0.0201 (15)	0.0024 (12)	−0.0013 (12)	−0.0036 (13)
N9	0.0144 (14)	0.0140 (13)	0.0215 (17)	0.0008 (10)	−0.0012 (13)	−0.0015 (12)
N10	0.0155 (14)	0.0193 (15)	0.0264 (17)	−0.0019 (11)	0.0007 (13)	0.0040 (13)
N11	0.0181 (14)	0.0146 (14)	0.0300 (17)	−0.0034 (11)	−0.0031 (13)	0.0001 (13)
C12	0.0163 (16)	0.0117 (15)	0.0184 (17)	0.0008 (13)	0.0031 (13)	0.0008 (13)
C13	0.0179 (16)	0.0139 (16)	0.021 (2)	0.0000 (13)	0.0011 (13)	−0.0009 (13)
C14	0.0185 (17)	0.0185 (17)	0.0208 (17)	0.0011 (14)	−0.0004 (14)	−0.0011 (14)
C15	0.0159 (16)	0.0183 (16)	0.0240 (17)	−0.0015 (13)	0.0026 (15)	0.0017 (15)
C16	0.0148 (16)	0.0144 (16)	0.0271 (19)	0.0005 (13)	0.0010 (14)	0.0001 (14)
C17	0.0168 (16)	0.0201 (17)	0.030 (2)	−0.0018 (13)	−0.0017 (17)	−0.0027 (15)
C18	0.0177 (17)	0.028 (2)	0.032 (2)	−0.0019 (15)	−0.0096 (16)	−0.0046 (16)
C19	0.0255 (19)	0.0251 (18)	0.019 (2)	0.0005 (14)	−0.0039 (15)	−0.0043 (15)
C20	0.0170 (15)	0.0155 (15)	0.0189 (17)	0.0025 (12)	−0.0026 (15)	0.0000 (14)
C21	0.0166 (16)	0.0177 (17)	0.0157 (17)	−0.0014 (13)	0.0016 (13)	−0.0010 (13)
C22	0.0210 (18)	0.0178 (18)	0.0250 (19)	−0.0036 (14)	−0.0055 (15)	−0.0023 (15)
C23	0.0178 (17)	0.0212 (17)	0.028 (2)	−0.0071 (14)	−0.0022 (15)	0.0033 (15)
C24	0.024 (2)	0.051 (3)	0.029 (2)	−0.0048 (18)	−0.0045 (19)	0.009 (2)
C25	0.020 (2)	0.079 (4)	0.041 (3)	0.003 (2)	−0.007 (2)	0.021 (3)
C26	0.018 (2)	0.058 (3)	0.040 (3)	0.0034 (19)	0.0026 (17)	0.022 (2)
C27	0.0155 (17)	0.032 (2)	0.036 (2)	0.0005 (15)	0.0050 (16)	0.0099 (18)
C28	0.0174 (15)	0.0158 (17)	0.0269 (19)	0.0007 (13)	0.0035 (14)	−0.0042 (15)
C29	0.0185 (16)	0.0148 (17)	0.0230 (19)	−0.0036 (13)	−0.0030 (14)	−0.0037 (14)
C30	0.0223 (19)	0.022 (2)	0.034 (2)	0.0023 (15)	−0.0071 (16)	−0.0069 (17)
C31	0.030 (2)	0.0183 (18)	0.042 (3)	−0.0002 (16)	−0.0142 (18)	0.0007 (17)
C32	0.029 (2)	0.022 (2)	0.036 (2)	−0.0088 (16)	−0.0109 (18)	0.0065 (18)
C33	0.0226 (18)	0.0234 (19)	0.029 (2)	−0.0073 (15)	−0.0052 (16)	0.0052 (16)

### (µ2-7-{[Bis(pyridin-2-ylmethyl)amino-1κ3N,N',N'']methyl}-5-chloroquinolin-8-olato-2κN;1:2κ2O)trichlorido-1κCl,2κ2Cl-dizinc(II) Geometric parameters (Å, º)

**Table:**

Zn1—Cl3	2.2190 (10)	C17—H17	0.9500
Zn1—Cl4	2.2241 (10)	C17—C18	1.360 (7)
Zn1—O7	2.019 (3)	C18—H18	0.9500
Zn1—N8	2.101 (3)	C18—C19	1.403 (6)
Zn2—Cl5	2.2897 (11)	C19—H19	0.9500
Zn2—O7	2.026 (3)	C21—H21A	0.9900
Zn2—N9	2.216 (3)	C21—H21B	0.9900
Zn2—N10	2.083 (3)	C22—H22A	0.9900
Zn2—N11	2.052 (3)	C22—H22B	0.9900
Cl6—C15	1.750 (4)	C22—C23	1.511 (5)
O7—C12	1.341 (4)	C23—C24	1.391 (6)
N8—C19	1.315 (5)	C24—H24	0.9500
N8—C20	1.358 (5)	C24—C25	1.387 (7)
N9—C21	1.487 (4)	C25—H25	0.9500
N9—C22	1.474 (5)	C25—C26	1.388 (8)
N9—C28	1.474 (4)	C26—H26	0.9500
N10—C23	1.335 (6)	C26—C27	1.377 (6)
N10—C27	1.344 (5)	C27—H27	0.9500
N11—C29	1.340 (5)	C28—H28A	0.9900
N11—C33	1.343 (5)	C28—H28B	0.9900
C12—C13	1.383 (5)	C28—C29	1.511 (5)
C12—C20	1.437 (5)	C29—C30	1.398 (5)
C13—C14	1.410 (5)	C30—H30	0.9500
C13—C21	1.505 (5)	C30—C31	1.374 (6)
C14—H14	0.9500	C31—H31	0.9500
C14—C15	1.370 (5)	C31—C32	1.390 (7)
C15—C16	1.409 (6)	C32—H32	0.9500
C16—C17	1.417 (5)	C32—C33	1.382 (6)
C16—C20	1.425 (5)	C33—H33	0.9500
			
Cl3—Zn1—Cl4	121.76 (4)	N8—C19—C18	122.3 (4)
O7—Zn1—Cl3	116.11 (8)	N8—C19—H19	118.8
O7—Zn1—Cl4	116.86 (8)	C18—C19—H19	118.8
O7—Zn1—N8	80.68 (11)	N8—C20—C12	117.1 (3)
N8—Zn1—Cl3	103.09 (9)	N8—C20—C16	121.5 (3)
N8—Zn1—Cl4	107.56 (9)	C16—C20—C12	121.5 (3)
O7—Zn2—Cl5	94.38 (8)	N9—C21—C13	113.5 (3)
O7—Zn2—N9	87.24 (10)	N9—C21—H21A	108.9
O7—Zn2—N10	123.92 (11)	N9—C21—H21B	108.9
O7—Zn2—N11	102.47 (11)	C13—C21—H21A	108.9
N9—Zn2—Cl5	176.25 (8)	C13—C21—H21B	108.9
N10—Zn2—Cl5	98.31 (10)	H21A—C21—H21B	107.7
N10—Zn2—N9	77.99 (12)	N9—C22—H22A	109.8
N11—Zn2—Cl5	103.95 (10)	N9—C22—H22B	109.8
N11—Zn2—N9	78.95 (12)	N9—C22—C23	109.2 (3)
N11—Zn2—N10	126.31 (12)	H22A—C22—H22B	108.3
Zn1—O7—Zn2	112.72 (12)	C23—C22—H22A	109.8
C12—O7—Zn1	114.1 (2)	C23—C22—H22B	109.8
C12—O7—Zn2	129.8 (2)	N10—C23—C22	116.3 (3)
C19—N8—Zn1	129.3 (3)	N10—C23—C24	121.5 (4)
C19—N8—C20	119.8 (3)	C24—C23—C22	122.2 (4)
C20—N8—Zn1	110.9 (2)	C23—C24—H24	121.1
C21—N9—Zn2	109.7 (2)	C25—C24—C23	117.9 (5)
C22—N9—Zn2	104.2 (2)	C25—C24—H24	121.1
C22—N9—C21	108.7 (3)	C24—C25—H25	119.7
C22—N9—C28	111.5 (3)	C24—C25—C26	120.6 (5)
C28—N9—Zn2	109.0 (2)	C26—C25—H25	119.7
C28—N9—C21	113.3 (3)	C25—C26—H26	121.1
C23—N10—Zn2	115.3 (2)	C27—C26—C25	117.9 (4)
C23—N10—C27	120.2 (4)	C27—C26—H26	121.1
C27—N10—Zn2	124.3 (3)	N10—C27—C26	121.9 (4)
C29—N11—Zn2	116.4 (3)	N10—C27—H27	119.0
C29—N11—C33	119.7 (3)	C26—C27—H27	119.0
C33—N11—Zn2	122.9 (3)	N9—C28—H28A	109.3
O7—C12—C13	124.5 (3)	N9—C28—H28B	109.3
O7—C12—C20	117.2 (3)	N9—C28—C29	111.7 (3)
C13—C12—C20	118.3 (3)	H28A—C28—H28B	107.9
C12—C13—C14	120.2 (3)	C29—C28—H28A	109.3
C12—C13—C21	122.0 (3)	C29—C28—H28B	109.3
C14—C13—C21	117.8 (3)	N11—C29—C28	118.8 (3)
C13—C14—H14	119.3	N11—C29—C30	120.9 (4)
C15—C14—C13	121.4 (4)	C30—C29—C28	120.2 (3)
C15—C14—H14	119.3	C29—C30—H30	120.4
C14—C15—Cl6	119.3 (3)	C31—C30—C29	119.3 (4)
C14—C15—C16	121.3 (3)	C31—C30—H30	120.4
C16—C15—Cl6	119.4 (3)	C30—C31—H31	120.2
C15—C16—C17	125.8 (3)	C30—C31—C32	119.7 (4)
C15—C16—C20	117.2 (3)	C32—C31—H31	120.2
C17—C16—C20	116.9 (4)	C31—C32—H32	120.9
C16—C17—H17	120.1	C33—C32—C31	118.2 (4)
C18—C17—C16	119.9 (4)	C33—C32—H32	120.9
C18—C17—H17	120.1	N11—C33—C32	122.3 (4)
C17—C18—H18	120.2	N11—C33—H33	118.8
C17—C18—C19	119.6 (4)	C32—C33—H33	118.8
C19—C18—H18	120.2		
			
Zn1—O7—C12—C13	177.9 (3)	C14—C15—C16—C20	0.2 (5)
Zn1—O7—C12—C20	−3.2 (4)	C15—C16—C17—C18	−177.9 (4)
Zn1—N8—C19—C18	−178.3 (3)	C15—C16—C20—N8	178.8 (3)
Zn1—N8—C20—C12	−1.1 (4)	C15—C16—C20—C12	−1.9 (5)
Zn1—N8—C20—C16	178.2 (3)	C16—C17—C18—C19	−0.3 (6)
Zn2—O7—C12—C13	20.3 (5)	C17—C16—C20—N8	0.6 (5)
Zn2—O7—C12—C20	−160.7 (2)	C17—C16—C20—C12	179.9 (3)
Zn2—N9—C21—C13	67.5 (3)	C17—C18—C19—N8	−0.2 (6)
Zn2—N9—C22—C23	43.1 (3)	C19—N8—C20—C12	179.6 (3)
Zn2—N9—C28—C29	21.0 (3)	C19—N8—C20—C16	−1.1 (5)
Zn2—N10—C23—C22	5.3 (4)	C20—N8—C19—C18	0.9 (6)
Zn2—N10—C23—C24	−176.1 (3)	C20—C12—C13—C14	−1.5 (5)
Zn2—N10—C27—C26	175.4 (3)	C20—C12—C13—C21	179.9 (3)
Zn2—N11—C29—C28	−8.9 (4)	C20—C16—C17—C18	0.1 (5)
Zn2—N11—C29—C30	167.5 (3)	C21—N9—C22—C23	−73.8 (4)
Zn2—N11—C33—C32	−167.5 (3)	C21—N9—C28—C29	143.5 (3)
Cl6—C15—C16—C17	−0.8 (5)	C21—C13—C14—C15	178.5 (3)
Cl6—C15—C16—C20	−178.8 (3)	C22—N9—C21—C13	−179.2 (3)
O7—C12—C13—C14	177.5 (3)	C22—N9—C28—C29	−93.5 (4)
O7—C12—C13—C21	−1.2 (6)	C22—C23—C24—C25	179.1 (4)
O7—C12—C20—N8	2.8 (5)	C23—N10—C27—C26	0.1 (6)
O7—C12—C20—C16	−176.4 (3)	C23—C24—C25—C26	−0.5 (8)
N9—C22—C23—N10	−34.8 (4)	C24—C25—C26—C27	0.2 (8)
N9—C22—C23—C24	146.6 (4)	C25—C26—C27—N10	0.0 (7)
N9—C28—C29—N11	−9.5 (4)	C27—N10—C23—C22	−179.0 (3)
N9—C28—C29—C30	174.0 (3)	C27—N10—C23—C24	−0.4 (6)
N10—C23—C24—C25	0.6 (7)	C28—N9—C21—C13	−54.6 (4)
N11—C29—C30—C31	0.8 (6)	C28—N9—C22—C23	160.6 (3)
C12—C13—C14—C15	−0.2 (6)	C28—C29—C30—C31	177.2 (3)
C12—C13—C21—N9	−48.1 (5)	C29—N11—C33—C32	0.3 (6)
C13—C12—C20—N8	−178.1 (3)	C29—C30—C31—C32	0.1 (6)
C13—C12—C20—C16	2.6 (5)	C30—C31—C32—C33	−0.8 (6)
C13—C14—C15—Cl6	179.9 (3)	C31—C32—C33—N11	0.6 (6)
C13—C14—C15—C16	0.9 (6)	C33—N11—C29—C28	−177.5 (3)
C14—C13—C21—N9	133.2 (3)	C33—N11—C29—C30	−1.0 (5)
C14—C15—C16—C17	178.2 (3)		

### (µ2-7-{[Bis(pyridin-2-ylmethyl)amino-1κ3N,N',N'']methyl}-5-chloroquinolin-8-olato-2κN;1:2κ2O)trichlorido-1κCl,2κ2Cl-dizinc(II) Hydrogen-bond geometry (Å, º)

**Table:**

*D*—H···*A*	*D*—H	H···*A*	*D*···*A*	*D*—H···*A*
C18—H18···Cl4^i^	0.95	2.77	3.537 (4)	139
C21—H21*B*···Cl4^ii^	0.99	2.69	3.584 (4)	150
C26—H26···Cl3^iii^	0.95	2.83	3.657 (4)	146
C28—H28*B*···Cl3^iv^	0.99	2.77	3.507 (4)	132

Symmetry codes: (i) −*x*+1, −*y*+1, *z*; (ii) *x*, *y*, *z*−1; (iii) −*x*+1/2, −*y*+1, *z*−1/2; (iv) −*x*+3/4, *y*−1/4, *z*−3/4.
